# Experimental analysis of time difference of arrival estimates based on inexactly reconstructed signals

**DOI:** 10.1038/s41598-025-24556-w

**Published:** 2025-11-19

**Authors:** Shanhe Wang, Changjiang Huang, Yu Xiang, Yuanyuan Gao, Xian Zhao, Yu Hua

**Affiliations:** 1 National Time Service Center, Chinese Academy of Sciences, Xi’an, 710600 Shaanxi China; 2 University of Chinese Academy of Sciences, Beijing, 100190 Beijing China

**Keywords:** Time difference of arrival, Compressed sensing, Cross-correlation, Engineering, Mathematics and computing, Optics and photonics

## Abstract

Abstract: Time Difference of Arrival (TDOA) estimation is a pivotal technique with extensive applications across various domains, including passive detection and indoor positioning. For signals characterized by unknown modulation types and originating from non-cooperative sources, achieving high-precision TDOA estimation traditionally necessitates a substantial volume of sampling data. Conventional approaches, such as cross-correlation, require high sampling rates and extended durations, posing significant challenges in terms of data acquisition, transmission, and storage. To surmount these obstacles, this paper delves into an enhanced inexact reconstruction-based compressed sensing method for TDOA estimation (EIRCS), presents an optimized algorithmic procedure that further reduces the computational complexity of the EIRCS method. Experimental results substantiate that the EIRCS method is an unbiased estimation technique. It accomplishes high-precision TDOA estimation concurrent with efficient data compression. These insights suggest that the EIRCS method can yield dependable TDOA estimates with minimal error, even at elevated compression ratios. Its capacity to sustain high accuracy across a range of compression ratios renders it particularly apt for applications demanding efficient data processing and reliable TDOA estimation.

## Introduction

In the realm of signal processing, the precise measurement of the time difference of arrival (TDOA) between a signal and its reflected counterpart, or between the same signal arriving at two distinct receiving devices, holds significant application value. For instance, in fields such as passive localization, indoor positioning, and the localization of capsules within the human gastrointestinal tract, TDOA serves as a crucial metric for achieving accurate positioning^1–5^. When other conditions are held constant, the higher the precision of TDOA estimation, the greater the accuracy of the positioning.

The cross - correlation method is a classic method for computing TDOA^6^. It determines TDOA by calculating the cross - correlation function of two signals and finding the delay value that maximizes the cross - correlation. However, this method has high requirements for signal bandwidth and signal-to-noise ratio (SNR). Otherwise, the wider the correlation peak, the more difficult it is to improve the estimation accuracy of TDOA. Many improved algorithms have been derived from the cross - correlation method, such as the generalized cross - correlation method and the adaptive filtering algorithm^7–11^. These methods use fixed or adaptive filters to preprocess the signal and suppress the interference of noise on the correlation peak, thereby improving the estimation accuracy of TDOA. These methods require certain prior knowledge of the signal to design reasonable filters or iterative step lengths.

Maximum likelihood estimation (MLE) estimates TDOA parameters by maximizing the likelihood function^12,13^. MLE can provide very accurate TDOA estimates, but it has high computational complexity and is highly dependent on model assumptions, which limits its use in some application scenarios.

In the frequency domain or other transform spaces, there are methods such as the power spectrum phase method, wavelet transform method, and subspace methods.

The power spectrum phase method transforms the peak detection of the cross - correlation function into the estimation of time difference using the phase function of the power spectrum^14,15^. However, phase ambiguity may occur when the estimated time difference is large. In such cases, prior knowledge is needed to correct the phase. For example, in the very long baseline interferometry (VLBI) measurement technique, after compensating for the delay according to the delay model, the slope of the phase and frequency of the power spectrum is calculated, which is the estimated value of TDOA.

The wavelet transform method performs wavelet decomposition and denoising of the signal under set parameters such as wavelet basis, decomposition level, and threshold, and then calculates the correlation. The performance of this method depends on the selection of wavelet basis and other parameters^16,17^.

Subspace methods, such as the Multiple Signal Classification(MUSIC) method, use the orthogonally of the signal subspace and noise subspace^18,19^. They estimate the parameter subspace by minimizing the projection of the noise subspace onto the signal matrix. In high SNR and large - sample cases, this method can provide very accurate TDOA estimates. However, its performance is highly dependent on the assumptions of the signal model and noise distribution, and it has high computational complexity.

In recent years, Compressed Sensing (CS) has emerged as a novel signal processing technique, widely employed to reduce the volume of sampled data while maintaining the precision of signal reconstruction. In Compressed Sensing, the compression sampling process involves using a measurement matrix to project the signal from a high - dimensional space to a low - dimensional space. Subsequently, the original signal is reconstructed by selecting an appropriate reconstruction algorithm based on the projection method. The sparse representation of the signal is a fundamental prerequisite for Compressed Sensing. That is to say, under a certain transform basis, the signal can be equivalently or approximately transformed into a sparse signal.

Suppose that a one - dimensional discrete signal of length *N*, denoted as $\varvec{s}$, is non - sparse. Under a certain basis transformation, it can be represented by a sparse signal $\varvec{\theta }$. Then,

1 $$\begin{aligned} \varvec{s} = \varvec{\Psi \theta }, \end{aligned}$$

where $\varvec{\Psi }_{N\times N}$ is the projection matrix, $\varvec{\theta } = [\theta _1, \theta _2, \cdots , \theta _N]^\textrm{T}$. If $\varvec{\theta }$ is *K*-sparse (i.e., the number of non-zero elements in $\varvec{\theta }$ is *K*), then $\varvec{\theta }$ is referred to as the *K*-sparse representation of the signal $\varvec{s}$.

Through a given $M \times N (M < N)$ measurement matrix $\varvec{\Phi }$, one can obtain the original signal $\varvec{s}$’s linear measurement value $\varvec{y}$,

2 $$\begin{aligned} \varvec{y} = \varvec{\Phi s} = \varvec{\Phi \Psi \theta } = \varvec{A \theta }, \end{aligned}$$

where $\varvec{A}$ is an $M \times N$ matrix, referred to as the sensing matrix.

In order to reconstruct sparse signals, the sensing matrix $\varvec{A}$ must satisfy the Restricted Isometry Property (RIP)^20^. That is, for any *K*-sparse vector $\varvec{\theta }$, there exists a constant $\delta _K> 0$, such that,

3 $$\begin{aligned} (1 - \delta _K) \Vert \varvec{\theta }\Vert _2^2 \le \Vert \varvec{A\theta }\Vert _2^2 \le (1 + \delta _K) \Vert \varvec{\theta }\Vert _2^2. \end{aligned}$$

If Eq (3) is not satisfied, it may transform two different *K*-sparse vectors into the same measurement value $\varvec{y}$ under the projection of $\varvec{A}$, thus making it impossible to accurately reconstruct. Some common measurement matrices that can make the sensing matrix satisfy the RIP condition include Gaussian random matrices, random Bernoulli matrices, partial Fourier matrices, sparse random matrices, and Toeplitz matrices, etc.^21^.

The sensing matrix $\varvec{A}$ is a matrix with far fewer rows than columns, and Eq (2) is an underdetermined system, which has infinitely many solutions. However, due to the prior condition that $\varvec{\theta }$ is a sparse vector, and the matrix $\varvec{A}$ has the RIP property, this guarantees that Eq (2) has a unique solution^22^. Common reconstruction methods include greedy algorithms, convex optimization, and Bayesian algorithms^21^.

TDOA estimation methods based on compressed sensing, as compared to traditional methods such as cross-correlation, can effectively reduce the amount of sampled data, thereby enabling efficient data transmission and storage. Numerous scholars have investigated TDOA estimation algorithms based on CS. These algorithms can be fundamentally categorized into two groups based on whether the compressed sampled signal $\varvec{y}$ is reconstructed.

1. Reconstruction: The basic procedure involves cascading existing CS and cross-correlation techniques. This involves designing a projection matrix based on the received signals to ensure that the signals have a sparse representation, selecting an appropriate measurement matrix to sample the signals, reconstructing the original signals from $\varvec{A}$ and $\varvec{y}$, and subsequently estimating TDOA through cross-correlation or generalized correlation operations.
2. Non-reconstruction: One approach is to use the fact that when the measurement matrix $\varvec{\Phi }$ is a Hadamard matrix, the compressed sampling results still preserve the temporal relationships of the original signals^23^. By correlating the compressed sampled signals $\varvec{y_1}$ and $\varvec{y_2}$, an estimate of TDOA can be obtained. Another method involves recognizing the proportional relationship between the phase of the product of the Discrete Fourier Transform (DFT) of $\varvec{y_1}$ and $\varvec{y_2}$ and TDOA, which allows for TDOA estimation^12^. There is also a method that samples one signal at the Nyquist rate, delays it by a certain time $\tau$, projects it through the measurement matrix to obtain the measurement result $\varvec{y_1}$, and then correlates it with the compressed sampled signal $\varvec{y_2}$ to calculate TDOA based on the peak value^24^.

In our preliminary research, we initially explored the feasibility of TDOA estimation using inexact reconstruction and proposed a fundamental inexact reconstruction method (IRCS)^25^. However, this method still has limitations in certain scenarios, particularly under high compression ratio, that is, when the number of rows in the measurement matrix is relatively small. To overcome these limitations, we have improved upon the IRCS method and introduced the enhanced inexact reconstruction-based compressed sensing method for TDOA estimation(EIRCS).

The EIRCS method employs the Orthogonal Matching Pursuit (OMP) algorithm for inexact reconstruction based on $\varvec{y}$ and $\varvec{A}$^26^. Although the reconstructed signal may differ significantly from the original signal, it retains the phase relationships between the original signals in the frequency domain, thus ensuring the accuracy of TDOA estimation. The focus of this method is on the precision of delay estimation rather than the accuracy of reconstruction, and it does not require the signal to have a sparse representation. Experimental validation has shown that the EIRCS method not only effectively reduces the amount of sampled data but also maintains high-precision TDOA estimation even under high compression ratio.

This study, by integrating the principles of the OMP algorithm and the particularities of matrix $\varvec{A}$, presents an optimized algorithmic process that effectively reduces the computational complexity of the EIRCS method. Meanwhile, this study investigates the performance of the EIRCS method for TDOA estimation across different compression ratio. Practical test data demonstrate that the EIRCS method exhibits excellent TDOA estimation performance, with promising application prospects.

## Methods

Assuming that the TDOA between the signal from the transmission or reflection point to the two sensors *D* is constant over the observation interval, the signal model received by Sensor 1 and Sensor 2 is represented as

4 $$\begin{aligned} {x_1}(n)&= s(n) + {n_1}(n)\nonumber \\ {x_2}(n)&= s(n -D ) + {n_2}(n) \end{aligned}$$

where *s*(*n*) is a stationary band-limited zero-mean random signal and the noises ${n_1}(n)$ and ${n_2}(n)$ are uncorrelated zero-mean white noise. $n = 1,2,...,N$, *N* represents the number of sampling points.

Rewrite Eq (4) in vector form, and after undergoing compression sampling by $\varvec{\Phi }_{M\times N}$, we obtain

5 $$\begin{aligned} \varvec{y_1}&=\varvec{\Phi x_1} = \varvec{\Phi }(\varvec{s_1} + \varvec{n_1})\nonumber \\ \varvec{y_2}&=\varvec{\Phi x_2} = \varvec{\Phi } (\varvec{s_2} +\varvec{ n_2}) \end{aligned}$$

where $\varvec{x_i} = [x_{i}(1), x_{i}(2), \cdots , x_{i}(N)]^\textrm{T}$, $\varvec{n_i} = [n_{i}(1), n_{i}(2), \cdots , n_{i}(N)]^\textrm{T}$, $i = 1, 2$, $\varvec{s_1} = [s(1),$ $s(2), \cdots , s(N)]^\textrm{T}$, and $\varvec{s_2}$ is the vector $\varvec{s_1}$ that is delayed or advanced by |*D*| sampling points. The superscript $\textrm{T}$ indicates the transpose of a vector.

Denote $\varvec{x} = \varvec{{s_1} + {n_1}}$, $\Delta \varvec{{n_{21}}} = \varvec{ {n_2} - {n_1}}$. Selecting projection matrix $\varvec{\Psi }$ as the inverse of the Fourier transform matrix and the measurement matrix $\varvec{\Phi }$ as the partial Fourier transform matrix,

6 $$\begin{aligned} \varvec{y_1}&=\varvec{A \theta } \nonumber \\ \varvec{y_2}&= \varvec{A(\theta }e^{j\frac{2\pi D}{N}}+ \Delta \varvec{\theta _{21}}) \end{aligned}$$

where, $$\varvec{A} = \varvec{\Phi \Psi }$$, $$\varvec{x} = \varvec{\Psi \theta }$$, $$\Delta \varvec{n_{21}} = \varvec{\Psi }\Delta \varvec{\theta _{21}}$$.

The matrix $\varvec{\Phi }$ is composed of *M* row vectors extracted from the Fourier transform matrix, and the set of row indices is denoted as $\varvec{\kappa }$. Consequently, $\varvec{A }$ is a matrix formed by extracting the rows corresponding to $\varvec{\kappa }$ from an $N \times N$ identity matrix. If $\varvec{\kappa }$ represents the set of indices corresponding to the *M* largest components in the DFT of $\varvec{x_1 }$, then $\varvec{y_1 }$ retains the *M* largest frequency components of $\varvec{x_1 }$, and $\varvec{y_2 }$ is similarly constructed.

The OMP reconstruction algorithm is employed to reconstruct $\varvec{y_1 }$ and $\varvec{y_2 }$, with the number of iterations set to *M*. This process yields the reconstructed signals $\varvec{\hat{\theta }_1}$ and $\varvec{\hat{\theta }_2}$, and it is ensured that

7 $$\begin{aligned} \varvec{\hat{\theta }_2} = \varvec{\hat{\theta }_1}e^{j\frac{2\pi D}{N}} + \Delta \varvec{\hat{\theta }_{21}}, \end{aligned}$$

where the *k*-th ($k \in \varvec{\kappa }$) element of $\Delta \varvec{\hat{\theta }_{21}}$ is identical to the *k*-th element of $\Delta \varvec{ \theta _{21}}$. All other elements of $\Delta \varvec{\hat{\theta }_{21}}$ are zero^25^.

The cross-correlation function between signals $\varvec{x_1}$ and $\varvec{x_2}$ is

8 $$\begin{aligned} R_{{x_1}{x_2}}^{CS}(\tau )&= IFFT(\varvec{\hat{\theta }_1}\varvec{\hat{\theta }_2}^*e^{j\frac{2\pi \tau }{N}})\nonumber \\&= IFFT(\varvec{\hat{\theta }_1}\varvec{\hat{\theta }_1}^*e^{j\frac{2\pi (\tau - D)}{N}}) + IFFT(\varvec{\hat{\theta }_1}\Delta \varvec{\hat{\theta }_{21}}^*e^{j\frac{2\pi \tau }{N}}), \end{aligned}$$

where $IFFT(\cdot )$ stands for inverse Fourier transform, and ${}^*$ denotes the complex conjugate.

When $\tau = D$, the first term on the right side of Eq (8) reaches its maximum value. The second term on the right side is the result of the correlation between some components of $\varvec{x_1}$ and some components of the noise $\varvec{n_2 - n_1}$. Under the assumption that the signal and noise are uncorrelated, the expectation of this part is approximately 0. Therefore, the TDOA estimate value is

9 $$\begin{aligned} \hat{D} = \arg \{\max (|R_{{x_1}{x_2}}^{CS}(\tau ) |)\}. \end{aligned}$$

This method is called the enhanced inexact reconstruction-based compressed sensing method for TDOA estimation, abbreviated as EIRCS.

## Optimization of the algorithmic procedure

According to the analysis in Section 2, $\varvec{y_i}$ is an *M*-dimensional column vector composed of the *M* largest components extracted from the DFT of $\varvec{x_i}$, encompassing partial spectral information of $\varvec{x_i}$.

**Fig. 1:**
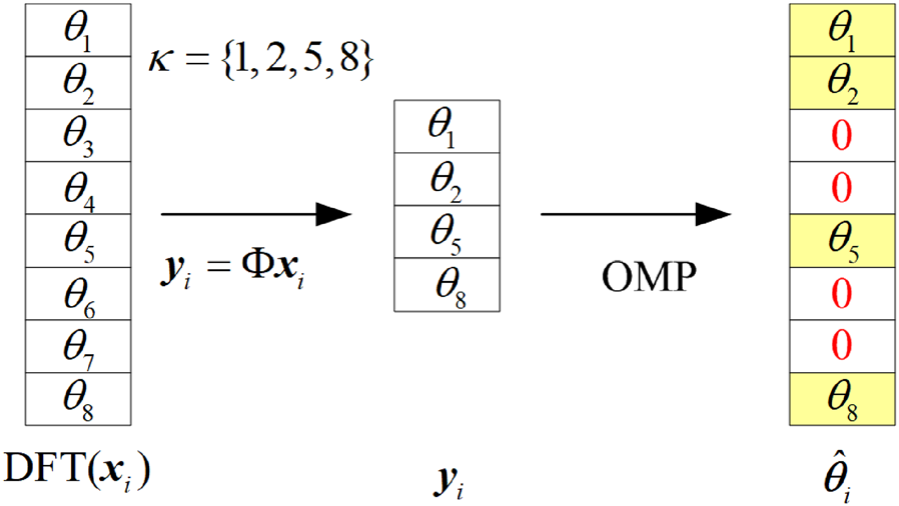
Schematic diagram of compressed sampling and reconstruction.

The OMP algorithm is a greedy algorithm that, at each iteration, identifies the atom from matrix $\varvec{A}$ that has the highest correlation with the current residual. Since $\varvec{A}$ is composed of $M$ rows extracted from an $N$-dimensional identity matrix, after the first iteration, the atom obtained is the element with the largest absolute value in $\varvec{y_i}$. The second iteration yields the atom that is the second largest in absolute value in $\varvec{y_i}$, and so on. After $M$ iterations, the reconstructed signal $\varvec{\hat{\theta }_i}$ is achieved by expanding $\varvec{y_i}$ into an $N$-dimensional column vector, where the elements of $\varvec{\hat{\theta }_i}$ corresponding to the indices in $\varvec{\kappa }$ takes on values from $\varvec{y_i}$, and the other elements are set to zero. As shown in Fig. 1, assuming $N=8$, $M=4$, and $\varvec{\kappa } = \{1, 2, 5, 8\}$.

Thus, the reconstruction result of the OMP algorithm is to assign the *j*-th element ($j = 1, \cdots M$) of $\varvec{y}$ to a certain element of an *N*-dimensional zero-value column vector, with the position of this element being $\varvec{\kappa }(j)$. This simple reconstruction result is completely consistent with the reconstruction result of the OMP algorithm. Therefore, the algorithmic process of EIRCS can be further optimized, conserving computational resources. Algorithm procedure:

- Set the signal sampling duration $T$ or sampling length $N$, the number of rows $M$ in the measurement matrix, and determine $\varvec{\kappa }$ based on the signal spectrum.
- Perform DFT on the sampled signals, retain the elements corresponding to the rows in $\varvec{\kappa }$, to obtain the measurement values $\varvec{y_1}$ and $\varvec{y_2}$.
- Based on $\varvec{\kappa }$, expand $\varvec{y_1}$ and $\varvec{y_2}$ to obtain the reconstructed $\varvec{\hat{\theta }_1}$ and $\varvec{\hat{\theta }_2}$.
- Estimate TDOA using Eq (9).

The computational complexity of the algorithm prior to optimization is primarily composed of two parts: the DFT and the OMP algorithm, which is approximately $O(N^2) + O(NMK)$. Since $M = K$, the complexity becomes $O(N^2) + O(NM^2)$. After optimization, the algorithm procedure avoids the computational process of the OMP algorithm, resulting in a reduced complexity of $O(N^2)$, effectively saving computational time. Particularly when *M* is relatively large, the optimized EIRCS algorithm will have a more evident reduction in time compared to the unoptimized version.

**Fig. 2:**
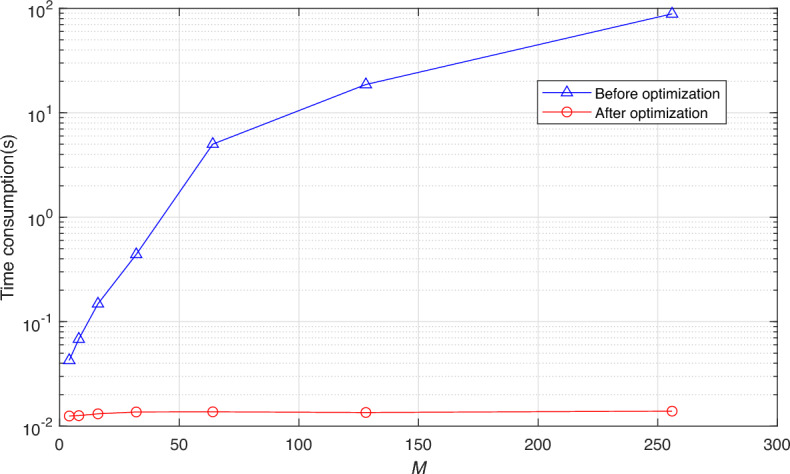
Time consumption corresponding to different values of *M* before and after optimization.

With *N* fixed at 1024 and *M* taking values of 4, 8, 16, 32, 64, 128, and 256 respectively. Figure 2 presents the time consumption by the EIRCS algorithm, both before and after optimization, over 100 runs for different values of *M*, with the unit being seconds. As can be observed from Fig. 2, when *M* is relatively small, such as 4 and 8, the time consumption by the EIRCS algorithm, whether optimized or not, is less than 0.1 seconds. As *M* increases, the time consumption by the unoptimized EIRCS algorithm increases exponentially, whereas the optimized algorithm is independent of *M*, with the time consumption consistently remaining between 0.1 and 0.2 seconds.

The EIRCS method is capable of functioning on existing sampling platforms to compress, reconstruct, and estimate TDOA of signals that have already been sampled. Additionally, it can be implemented on hardware platforms that support random sampling.

## Experiments

In this section, the TDOA is calculated for the same data using both the EIRCS method and the cross - correlation method. The estimation accuracy of the EIRCS method is verified by comparing the errors between the two methods. Additionally, the TDOA estimation errors of the EIRCS method under different compression ratios are compared.

### Composition of the experimental platform

**Fig. 3:**
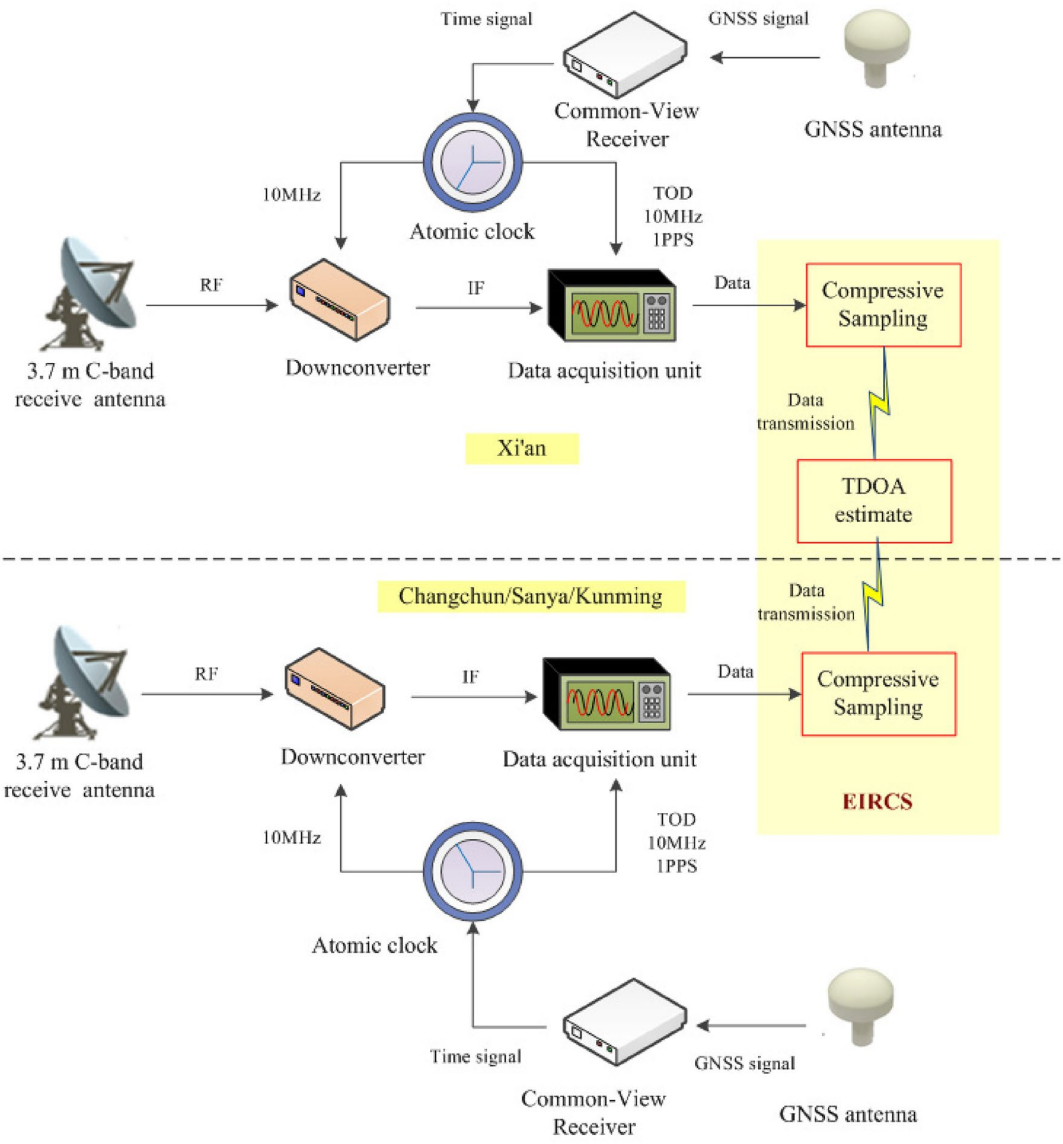
Schematic diagram of the experimental platform composition.

As shown in Fig. 3, the experimental platform consists of four ground stations located in Xi’an, Changchun, Sanya, and Kunming. Each ground station is composed of a 3.7-meter C-band receive antenna, a downconverter, a data acquisition unit, and a clock synchronization system.

The 3.7 - meter C - band receive antennas at each ground station conduct time - shared synchronous observations of three GEO satellites: Zhongxing - 12, Zhongxing - 10, and AsiaSat - 7. The observation target of the satellite is switched synchronously every half - hour, and the RF signal with a downlink frequency of 3826MHz is received.

The downconverter down - converts the RF signal to a 70MHz intermediate - frequency signal.

The data acquisition unit has a sampling rate of 187.5MHz and collects data every 10 minutes.

The clock synchronization system, composed of a satellite - common - view receiver and an atomic clock, synchronizes the time of each ground station with a precision better than 5ns.

With Xi’an station as the reference station, the ground stations in Changchun, Kunming, and Sanya utilize the observational data to respectively calculate the TDOA between each station and the reference station using both the EIRCS method and the cross - correlation method(Abbreviated as CC). The TDOA values obtained by these two methods are denoted as $\tau _{EIRCS}$ and $\tau _{CC}$, respectively. For the CC method, the number of data points *N* is 18750, corresponding to a sampling duration of 0.1 ms. For the EIRCS method, the number of rows in the measurement matrix $\varvec{\Phi }$ is 375, that is, $M = 375$. If the compression ratio is defined as

10 $$\begin{aligned} CR = \frac{N}{M}, \end{aligned}$$

then the compression ratio for the EIRCS method is 50.

**Fig. 4:**
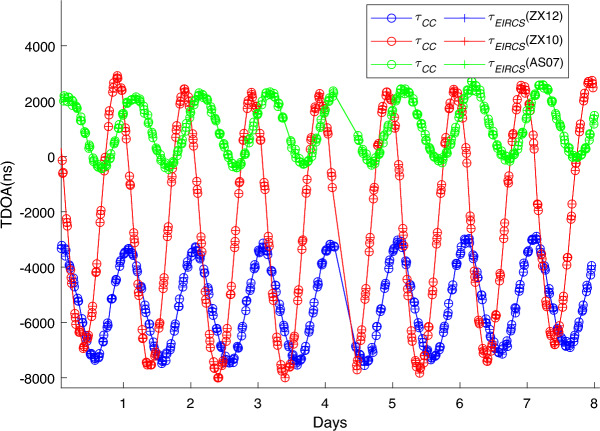
TDOA measurement values between Changchun station and the reference station for various satellites.

The experiment lasted for 8 days (with approximately 7 hours of equipment failure on Day 4, resulting in no data). Figure 4 presents the TDOA values between Changchun station and the reference station for Zhongxing - 12 (ZX12), Zhongxing - 10 (ZX10), and AsiaSat - 7 (AS07). The blue curve represents ZX12, the red curve represents ZX10, and the green curve represents AS07. The curves with circles denote the TDOA $\tau _{CC}$ between Changchun station and the reference station calculated using the CC method, while the curves with “+” signs denote the TDOA $\tau _{EIRCS}$ between Changchun station and the reference station calculated using the EIRCS method (with a compression ratio of 50). As can be seen from Fig. 4, on the scale of thousands of nanoseconds, $\tau _{EIRCS}$ and $\tau _{CC}$ are almost coincident.

### Analysis of the estimation performance of EIRCS

To further analyze the error between $\tau _{EIRCS}$ and $\tau _{CC}$, let

11 $$\begin{aligned} \Delta \tau = \tau _{EIRCS} - \tau _{CC}. \end{aligned}$$

**Fig. 5:**
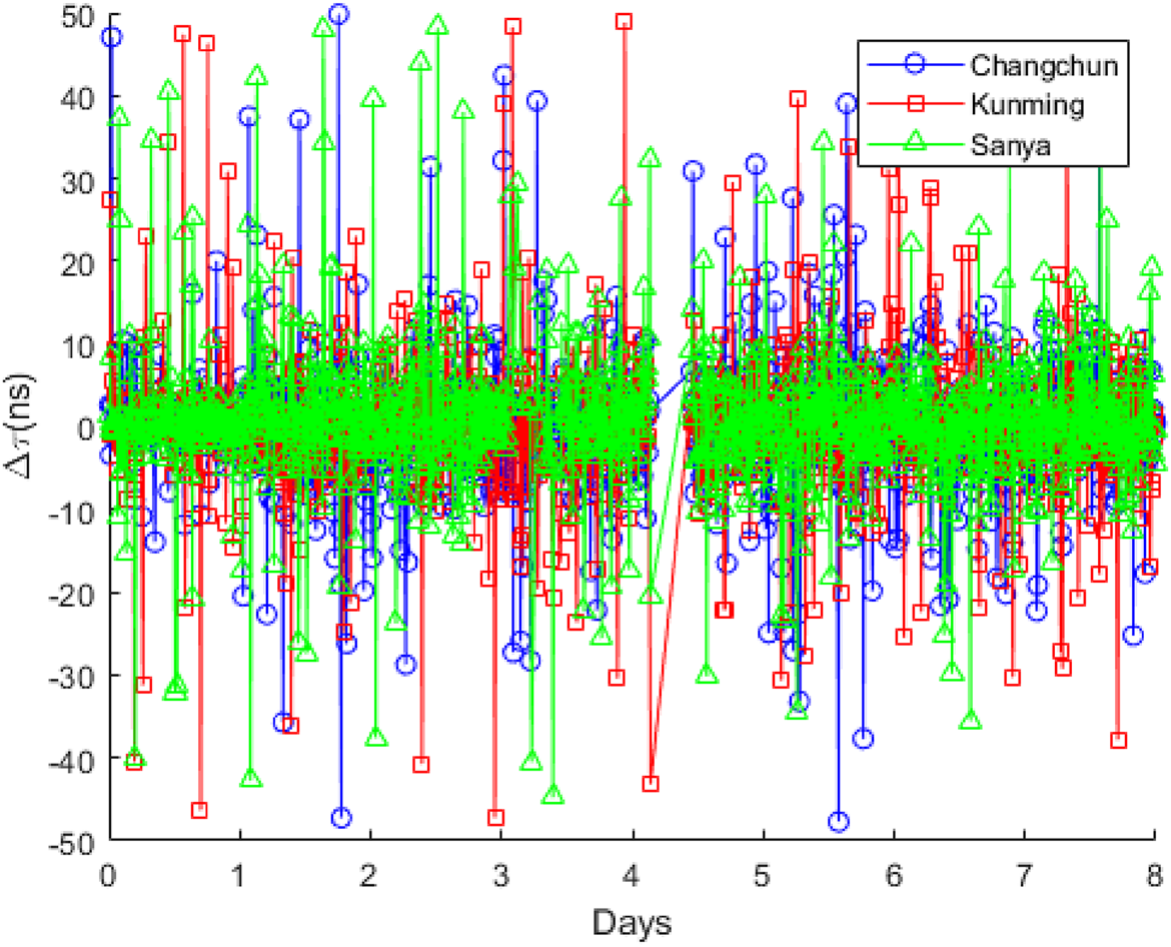
Calculation results of $\Delta \tau$ for Changchun, Kunming, and Sanya.

That is, $\Delta \tau$ represents the error of the TDOA calculated by the EIRCS method relative to that calculated by the CC method. Although the TDOA measurements corresponding to different satellites are discontinuous, $\Delta \tau$ is continuous. Figure 5 presents the calculation results of $\Delta \tau$ for Changchun, Kunming, and Sanya during the experimental period (with a compression ratio of the EIRCS method, $CR = 50$). The blue curve with circles corresponds to Changchun station, the red curve with squares corresponds to Kunming, and the green curve with triangles corresponds to Sanya.

**Fig. 6:**
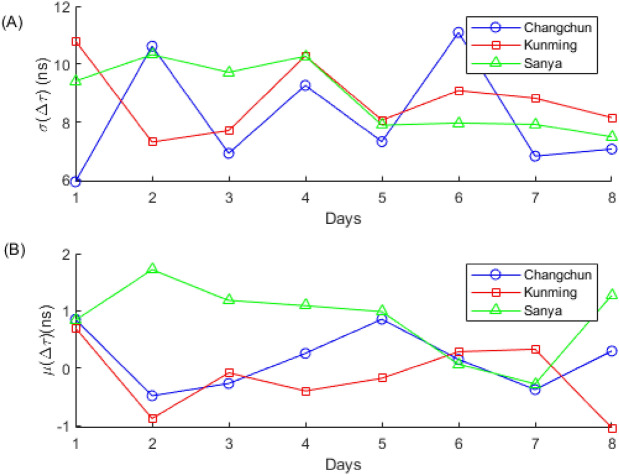
Statistical results of $\Delta \tau$. (**A**) The standard deviation of $\Delta \tau$. (**B**) The mean of $\Delta \tau$.

As can be seen from Fig. 5, despite the compression ratio $CR = 50$, $\Delta \tau$ is almost always within $\pm 50$ ns, with the majority falling within $\pm 10$ ns. Fig. 6 presents the statistical results of $\Delta \tau$. The statistics are calculated on a daily basis, including the standard deviation $\sigma (\Delta \tau )$ and the mean $\mu (\Delta \tau )$, as shown in Fig. 6(A) and (B), respectively.

As can be observed from Fig. 6, when the compression ratio $CR = 50$, the mean $\mu (\Delta \tau )$ of the three stations fluctuates within the range of $-1$ ns to 2 ns, indicating that the mean of $\tau _{EIRCS}$ is very close to the mean of $\tau _{CC}$. This suggests that the EIRCS method is an unbiased estimation method. The standard deviation $\sigma (\Delta \tau )$ fluctuates within the range of 6 ns to 1 ns. The $\sigma (\Delta \tau )$ consists of three components: 1) the standard deviation of the TDOA estimation by the EIRCS method, 2) the standard deviation of the TDOA estimation by the CC method, and 3) the standard deviation introduced by the time synchronization error. Therefore, the standard deviation of the TDOA estimation by the EIRCS method is better than $\sigma (\Delta \tau )$.

**Fig. 7:**
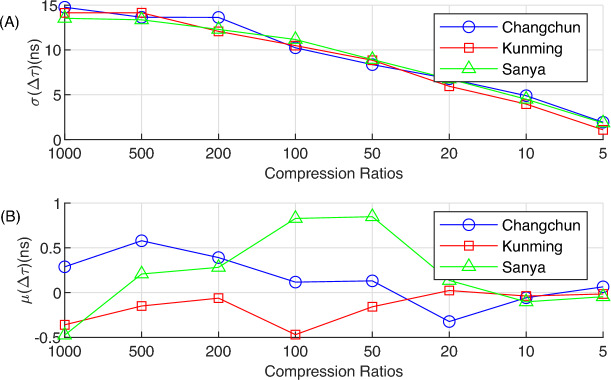
Statistical results of $\Delta \tau$ under different compression ratios. (**A**) The standard deviation of $\Delta \tau$. (**B**) The mean of $\Delta \tau$.

While keeping the sampling duration at 0.1 ms unchanged, the compression ratio, that is, the number of rows *M* of the measurement matrix $\varvec{\Phi }$, is varied. Under each compression ratio, the standard deviation $\sigma (\Delta \tau )$ and the mean $\mu (\Delta \tau )$ of all $\Delta \tau$ values over the 8 - day period are calculated, as shown in Fig. 7(A) and (B), respectively.

Figure 7(A) shows that when the compression ratio $CR \approx 1000$ (i.e., $M = 19$), the standard deviation $\sigma (\Delta \tau )$ of all three stations is around 15 ns. As the compression ratio decreases, $\sigma (\Delta \tau )$ shows a downward trend. This is because the data used gradually approaches the original data, and $\tau _{EIRCS}$ becomes closer to $\tau _{CC}$. When the compression ratio is 5, the $\sigma (\Delta \tau )$ of all three stations is around 2 ns.

Figure 7(B) shows that regardless of whether the compression ratio is 5 or 1000, the absolute value of the mean $|\mu (\Delta \tau )|$ of all three stations does not exceed 1 ns. Especially when the compression ratio is 5 or 10, $\mu (\Delta \tau )$ is around 0 ns, which further verifies that the EIRCS method is an unbiased estimation.

**Fig. 8:**
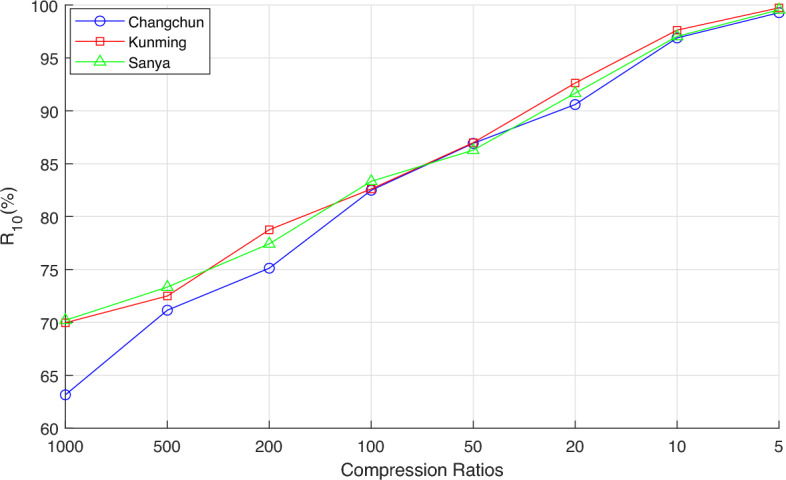
Statistical results of $R_{10}$ under different compression ratios.

Figure 8 statistically analyzes the probability that $\Delta \tau \le 10$ ns under different compression ratios, denoted as $R_{10}$. When the compression ratio is 1000, $R_{10}$ is in the range of $64\%$ to $70\%$, indicating that the difference $\Delta \tau$ between $\tau _{EIRCS}$ and $\tau _{CC}$ fluctuates significantly, and the estimation accuracy of $\tau _{EIRCS}$ is relatively low. This is also the reason why the corresponding $\sigma (\Delta \tau )$ is relatively large when the compression ratio is 1000 in Fig. 7(A). As the compression ratio decreases, $R_{10}$ gradually increases. When the compression ratio is 5, the $R_{10}$ of all three stations exceeds $99\%$.

## Conclusion

This study optimizes the algorithmic process of EIRCS, on this basis, evaluates the performance of the EIRCS method through an 8 - day experiment utilizing four ground stations located in Xi’an, Changchun, Sanya, and Kunming. The results demonstrate that the EIRCS method exhibits robust estimation performance across various compression ratios.

When the compression ratio is as high as 1000, the standard deviation $\sigma (\Delta \tau )$ of the TDOA estimation error $\Delta \tau$ is approximately 15 ns. As the compression ratio decreases to 5, $\sigma (\Delta \tau )$ decreases to around 2 ns. This indicates that the estimation accuracy of the EIRCS method significantly improves with the increase in the amount of data used, maintaining high precision even at higher compression ratios.

At a compression ratio of 5, the probability $R_{10}$ that $\Delta \tau$ is within $\pm 10$ ns exceeds $99\%$. This suggests that the EIRCS method can achieve high - precision TDOA estimation with a minimal error range at higher compression ratios.

In summary, the EIRCS method achieves high - precision TDOA estimation while maintaining efficient data compression, making it a reliable and efficient estimation approach. Its ability to maintain high accuracy even at high compression ratios renders it highly applicable in scenarios requiring efficient data processing and reliable TDOA estimation. Future research may focus on further optimizing the compression ratio for specific applications and exploring the potential of the EIRCS method in other signal processing domains.

## Data Availability

The datasets used and analysed during the current study available from the corresponding author on reasonable request.
